# Value of follow-up diagnostic radioiodine scans in differentiated thyroid cancer

**DOI:** 10.1530/EC-24-0007

**Published:** 2024-04-12

**Authors:** Teresa Kraus, Natalia Shengelia-de Lange, Holger Einspieler, Marcus Hacker, Alexander Haug, Elisabeth Kretschmer-Chott, Georgios Karanikas

**Affiliations:** 1 Division of Nuclear Medicine, Department of Biomedical Imaging and Image-Guided Therapy, Medical University of Vienna, Vienna, Austria; 2 Division of Nuclear Medicine, Tbilisi State Medical University, Tbilisi, Georgia

**Keywords:** thyroid cancer, thyroglobulin, follow-up, diagnostic radioiodine whole-body scan

## Abstract

**Background:**

The most important part of the follow-up of differentiated thyroid carcinoma (DTC) is the measurement of serum thyroglobulin (Tg). An increase of Tg levels indicates likely tumor recurrence. According to the guidelines of the European Society of Medical Oncology (ESMO), the follow-up should consist of serum Tg assays and a neck ultrasound, while the American Thyroid Association (ATA) recommends serum Tg assays, neck ultrasounds, and a diagnostic radioiodine whole-body scan (WBS) if non-stimulated Tg is greater than 10 ng/mL or if Tg is rising. This study questions the necessity of a diagnostic WBS in patients with low stimulated Tg levels during the initial follow-up.

**Design:**

This study is a retrospective data analysis.

**Methods:**

The data of 185 patients, who were in regular treatment and aftercare between 2015 and 2018 at the Department of Nuclear Medicine in Vienna, as well as the data of 185 patients who were treated in Tbilisi between 2015 and 2019, were analyzed.

**Results:**

There was a highly significant relationship between low stimulated Tg levels (<0.5 ng/mL) and the outcome of the diagnostic WBS at the first follow-up (*χ* ^2^ = 14.7, *P* < 0.001). In total, 31 out of 370 patients (8.4%) had positive findings in the diagnostic WBS. Seventy-five of 370 patients (19.74%) had stimulated Tg levels >0.5 ng/mL.

**Conclusion:**

Our data suggest that the first follow-up, 4–12 months after the initial therapy of DTC, including the measurement of basal and stimulated Tg levels and Tg antibody levels, does not mandate a diagnostic WBS on all patients.

**Significance statement:**

In this study, we examined the still commonly used routine diagnostic radioiodine whole-body scan in the first follow-up of patients with differentiated thyroid carcinoma. We questioned the necessity of the scan in patients with low stimulated thyroglobulin levels. Therefore, we combined retrospective data from the University Hospital in Vienna and in Tbilisi to analyze 370 patients. We were able to demostrate a highly significant relationship between low stimulated thyroglobulin levels (<0.5 ng/mL) and the outcome of the diagnostic scan at the first follow-up (*χ* = 14.7, *P* < 0.001).

## Introduction

Thyroid carcinoma (TC) is the most common endocrine malignancy, accounting for about 2.1% of all malignant diseases, with 84–94% being differentiated thyroid carcinoma (DTC) (1, 2, 3). After a patient receives treatment for DTC, which may include a total thyroidectomy and radioiodine remnant ablation, a follow-up visit is scheduled 4–12 months later. To determine the clinical outcome of the individual patient, several tests and examinations are performed. Thyroglobulin (Tg) is measured in the blood, as an increase of Tg over time is a strong indication that a relapse has occurred (4, 5, 6, 7, 8, 9). Another part of the follow-up is imaging methods, including an ultrasound or a diagnostic radioiodine whole-body scan (WBS) to possibly locate the area of recurrence.

According to the guidelines of the European Society of Medical Oncology (ESMO), the follow-up should consist of serum Tg assays, serum Tg antibodies, and a neck ultrasound to assess the response to the initial treatment 6–18 months after radioiodine ablation therapy. If there are any suspicious findings during the follow-up, such as rising serum Tg levels, the patient could be at risk for locoregional or distant metastases, and further imaging studies should be performed (10, 11).

The American Thyroid Association (ATA) states that a diagnostic WBS 1 year after radioiodine ablation is not required in low- and intermediate-risk patients. Instead, the ATA recommends serum Tg assays, serum Tg antibody levels, and neck ultrasounds at the first follow-up. For patients classified as high-risk patients or patients with intermediate risk (higher risk features) according to ATA criteria, and who typically have either a biochemical or structural incomplete response to therapy, a diagnostic WBS may have a role in the follow-up if non-stimulated Tg is greater than 10 ng/mL or if Tg levels are rising (12, 13, 14).

Some studies suggest that a diagnostic WBS in patients with low stimulated Tg levels (<2 ng/mL) is redundant (15, 16, 17, 18, 19). Other studies have demonstrated that the combination of a neck ultrasound and stimulated Tg levels can identify a relapse of DTC without the need for a diagnostic WBS (20, 21, 22).

To provide a clearer perspective in guidelines and literature regarding the clinical value of the diagnostic WBS in the follow-up, this study questions the still-common routine use of the scan in all patients with DTC in some practices.

## Materials and methods

### Patients

In this retrospective analysis, 185 patients (123 women, 62 men), aged 25–82 years (mean age: 52.9 years), who received follow-up after undergoing total thyroidectomy and radioiodine therapy due to DTC (which includes PTC and FTC) between 2015 and August 2018 at the Department of Nuclear Medicine at Allgemeines Krankenhaus (AKH) in Vienna, were included. The Ethics Committee of the Medical University of Vienna approved this retrospective study (EK Nr: 2058/2018). In addition, the data of 185 patients (159 women, 26 men), aged 15–70 years, who had the same follow-up after DTC therapy at the university hospital in Tbilisi, Georgia, between 2015 and January of 2019, were collected. An ethics committee vote is not required for a retrospective study in Georgia. This collected data showed no material differences in the distribution of findings and are adequately comparable. All patients enrolled in this study fulfilled the following criteria: histologically proven DTC; standard treatment of DTC consisting of total thyroidectomy followed by radioiodine therapy and TSH-suppressive thyroid hormone therapy; and complete follow-up examination 4–12 months after the standard treatment of DTC, which included the measurement of serum Tg levels, serum Tg antibodies, and a radioiodine WBS.

### Laboratory investigations

All patients who were followed up at the Medical University of Vienna had measurements of TSH, Tg, and Tg antibodies using the Immulite 2000 Anti-TPO (EURO/DPC, Gwynedd, UK). At the university hospital in Tbilisi, Georgia, the Firma Roche Tg Assay (with confirmation tests) was used for the measurement of TSH, Tg, and Tg antibodies. The laboratory results collected from both centers were compared and analyzed together. In Vienna, TSH was stimulated exogenously by administering recombinant human TSH (rhTSH) (Thyrogen 0.9 mg) intramuscularly for 2 days in a row. Stimulated Tg levels were measured on the 5th day after the first injection of rhTSH. In Georgia, TSH was stimulated endogenously through thyroid hormone withdrawal (THW) for 4–6 weeks.

Tg antibodies >115 IU/mL were considered positive, Tg antibodies between 33 and 115 IU/mL were categorized as the gray area, and Tg antibodies under 33 IU/mL were classified as negative.

### Imaging method

In Vienna, on the 3rd day after the first injection of rhTSH, 131-iodine (370 MBq) was administered orally, and the planar diagnostic WBS using the Symbia Intevo SPECT/CT was obtained 48–72 h later. The GE (Brivo NM 615) SPECT/CT was used for the planar diagnostic WBS in Tbilisi. To determine if there is pathological uptake present, the images were analyzed by two medical doctors in the field of nuclear medicine, with at least one of them being a board-certified specialist. Factors such as the characteristics of the uptake, including the presence of focal or diffuse uptake, abnormal distribution, or unexpected locations of iodine accumulation, as well as looking at uptake patterns that deviate from the expected physiological distribution, an informed assessment can be made regarding the pathological nature of the uptake observed in diagnostic radioiodine whole body scans.

### Statistical methods

Statistical analysis was performed using version IBM SPSS Statistics 25 of the statistical computing software SPSS. The analysis was carried out by means of descriptive statistics. An aim of this retrospective study was the estimation of the prevalence of positive diagnostic radioiodine WBS in patients with low stimulated serum Tg level. The percentage (95% Clopper–Pearson confidence interval) of these patients was calculated. For the main question, a McNemar test was used to analyze the statistical relationship between low stimulated Tg levels (<0.5 ng/mL) and the outcome of the diagnostic WBS at the first follow-up.

## Results

In total, 370 patients were observed in this two-center study. All patients had histologically proven DTC. Of the 370 patients, 282 were women (76%) and 88 were men (24%). The median age of included patients was 49 years, with 53 years being the median age in Vienna and 44 years in Tbilisi. The AJCC TNM clinical staging can be found in Table 1 and Supplementary Table 1 (see section on supplementary materials given at the end of this article). There was no statistically significant difference between the TNM staging in patients from Vienna and Tbilisi. The distribution of histotype showed 345 patients with papillary thyroid cancer (PTC) (93%) and 25 patients with follicular thyroid cancer (FTC) (7%) (Table 1).

**Table 1:** Clinicopathologic characteristics of included patients.

**Variable**	**Vienna (*n* = 185)**	**Georgia (*n* = 185)**	**Total (*n* = 370)**
Age	52.9 (25–82)	43.8 (15–70)	49 (15–82)
Gender			
Male	62 (34%)	26 (14%)	88 (24%)
Female	123 (66%)	159 (86%)	282 (76%)
TNM staging			
I	97 (51%)	96 (52%)	193 (52%)
II	33 (18%)	23 (12%)	56 (15%)
III	50 (27%)	65 (35%)	115 (31%)
IV	5 (3%)	1 (1%)	6 (2%)
Histology			
Papillary	160 (86%)	185 (100%)	345 (93%)
Follicular	25 (14%)	0 (0%)	25 (7%)

Values are expressed as median (range), number (%).

TNM, tumor node metastasis.

Patients received the diagnostic WBS 4–12 months after radioiodine ablation therapy. In Vienna, blood tests were performed after exogenous TSH stimulation, and in Tbilisi, after endogenous TSH stimulation with thyroid hormone withdrawal, before the diagnostic WBS. Two hundred ninety-five (78%) patients had stimulated Tg levels below 0.5 ng/mL, and 75 (20%) had stimulated Tg levels above 0.5 ng/mL. In 339 (92%) patients, the diagnostic WBS showed only physiological uptake of iodine-131, while 31 scans had positive results with pathological uptake (8%).

Tg antibodies were positive in 16 (4%) patients with levels above 115 IU/mL. There were 32 patients (9%) who had a low presence of Tg antibodies, between 33 and 115 IU/mL, while 322 (87%) showed an absence of Tg antibodies (Table 2).

**Table 2:** Results of the first follow-up.

**Variable**	**Vienna**(*n* = 185)	**Georgia**(*n* = 185)	**Total**(*n* = 370)
Stimulated Tg			
<0.5 ng/mL	145 (78%)	150 (81%)	295 (80%)
>0.5 ng/mL	40 (22%)	35 (19%)	75 (20%)
Diagnostic WBS			
Only physiological uptake	170 (92%)	169 (91%)	339 (92%)
Pathological uptake	15 (8%)	16 (9%)	31 (8%)
Tg antibodies			
Positive	7 (4%)	9 (5%)	16 (4%)
Gray area	10 (5%)	22 (12%)	32 (9%)
Negative	168 (91%)	154 (83%)	322 (87%)

Values are expressed as number (%).

Among the 31 patients who had a pathological uptake in the diagnostic WBS, 15 had stimulated Tg levels that were lower than 0.5 ng/mL, while the other 16 patients had levels above 0.5 ng/mL.

Of all the patients included in this study, 75% had no pathological uptake in the diagnostic WBS as well as stimulated Tg levels <0.5 ng/mL. There was a highly significant relationship between stimulated Tg levels and the result of the diagnostic WBS (*χ* ^2^ = 14.7, *P* < 0.001). The proportion of WBS-positive patients was higher in the patient group with Tg > 0.5 ng/mL than in the group with low Tg levels (<0.5 ng/mL) (Table 3).

**Table 3:** Relationship between Tg and WBS.

	**WBS positive**	**WBS negative**	***P***
Stimulated Tg			<0.001
>0.5 ng/mL	15 (20%)	60 (80%)	
<0.5 ng/mL	16 (5.4%)	279 (94.6%)	

Values are expressed as number (%). *P* > 0.05

Further analysis of the clinical pathology was conducted on the 15 patients with pathological findings on the WBS who underwent follow-up at the Medical University of Vienna. In previous check-ups 3 and 6 months after the radioiodine therapy, an ultrasound of the neck was performed. All 15 patients showed no indication of a local recurrence or residual thyroid tissue in the thyroid bed on both sides and no evidence of suspicious or pathological cervical lymph nodes in the neck ultrasound. The results of the analysis of the follow-up of these 15 patients are described in Table 4. Five of 15 (33.3%) had Tg levels under 0.5 ng/mL, while the other 10 patients had stimulated Tg levels ≥ 0.5 ng/mL. Tg antibodies were detectable in 20% of these ten patients, which is higher than in the general population of this study, where 13% of patients had detectable Tg antibodies. Nine (60%) of the positive WBS showed uptake in the thyroid bed, which could be due to residual thyroid tissue, two (13%) showed diffuse uptake in the general neck area, three (20%) showed uptake in the mediastinum, and one patient (7%) had uptake in the thyroid bed as well as the mediastinum. Of the 15 patients with pathological findings on the WBS, 12 required a second radioiodine therapy (80%).

**Table 4:** Summary of clinicopathologic data of the 15 patients with pathological uptake in the diagnostic WBS in Vienna.

**Patient**	**Age/gender**	**TNM staging**	**Unstimulated Tg**	**Stimulated Tg**	**Diagnostic WBS**	**TgAB**	**Second radioiodine therapy**
1	66/F	2	0.3	1.7	Mediastinum	N	N
2	61/M	1	0.09	0.4	Thyroid bed	N	Y
3	39/F	1	0.09	0.9	Thyroid bed	Y	Y
4	54/F	1	0.09	0.2	Thyroid bed	N	N
5	56/F	1	0.9	0.9	Thyroid bed	N	Y
6	54/F	3	0.09	3.1	Thyroid bed + mediastinum	Y	Y
7	37/F	1	0.5	9	Thyroid bed	N	Y
8	75/M	1	0.09	0.09	Mediastinum	N	N
9	81/F	1	0.09	0.4	Thyroid bed	N	Y
10	63/F	3	0.6	10.4	Thyroid bed	N	Y
11	76/F	1	0.09	0.09	Neck	Y	Y
12	80/F	1	0.8	12.3	Thyroid bed	N	Y
13	58/M	3	0.09	0.5	Neck	N	Y
14	40/F	1	2	6.9	Thyroid bed	N	Y
15	57/F	3	5.5	29.7	Mediastinum	N	Y

AB, antibody; F, female; M, male; N, no; Tg, thyroglobulin; TNM, tumor node metastasis; WBS, whole-body scan; Y, yes.

## Discussion

This study examined the results of the first follow-up at two centers in two different cities performed at 4–12 months in 370 patients diagnosed with DTC who were initially treated with a total thyroidectomy and radioiodine ablation. There was a highly statistically significant relationship between a low stimulated Tg level (<0.5 ng/mL) and no pathological uptake in the diagnostic WBS (*χ* ^2^ = 14.7, *P* < 0.001).

There were 339 patients who had a negative radioiodine WBS, which accounts for 92% of patients included in this study. In a study by Torlontano and coworkers, 100% of their 99 patients had a negative radioiodine WBS. However, it should be mentioned that those authors included only patients who were initially classified as low-risk patients. Their study concluded that a radioiodine WBS after rhTSH stimulation is useless in the first follow-up of DTC patients. They recommend performing an ultrasound examination of the neck, as they were able to identify lymph node metastases in two of the 78 patients who had negative Tg levels (<1 ng/mL) (23).

There are other studies that have reported that a radioiodine WBS is not necessary in patients with rhTSH-stimulated serum Tg concentrations that are <2 ng/mL (16, 24, 25).

The American Thyroid Association also lists in their guidelines for the treatment of DTC that a routine follow-up diagnostic WBS at the 1-year follow-up after radioiodine ablation therapy is not required in patients at low- and intermediate-risk (12, 26, 27, 28). This is in contrast to other guidelines, which recommend a WBS in all cases (29).

In our study, patients were included from all risk groups as negative Tg levels were analyzed at the 4–12 months follow-up, and the patient’s previous initial risk stratification was not taken into consideration. This enabled our study to determine the correlation between Tg levels measured at the 5th day of the follow-up and the outcome of the radioiodine WBS, while including patients who were previously defined as high-risk or intermediate-risk and who were excluded in other studies, as in the study by Torlontano and coworkers (23). A highly significant statistical relationship between the outcome of the radioiodine WBS and the Tg level was shown, questioning the necessity of the diagnostic radioiodine scan regardless of the risk group of the patient.

This study included data collected at two medical centers in two different countries, which allowed a greater number of patients to be included and provided the ability to compare results among centers. The analyzed results showed no material differences in the distribution of findings, indicating adequate comparability, which increases the generalizability of the study.

There are several studies that have compared a diagnostic WBS with an ultrasound of the neck (20, 28). A study by Torlontano and coworkers in 2006 with a cohort of 80 patients who had undergone near-total thyroidectomy without postoperative radioiodine ablation treatment found no pathological findings in the WBS in 100% of the patients. However, neck ultrasonography identified lymph node metastases in both Tg-positive (Tg >1 ng/mL) and Tg-negative patients (30).

Therefore, the first follow-up for patients 4–12 months after the treatment of DTC could consist of the measurement of basal and stimulated Tg levels and an ultrasound of the neck. If there are elevated Tg levels, further imaging methods, such as a diagnostic WBS, PET-CT, or non-contrast CT, are necessary. In patients with elevated Tg antibodies, further imaging methods should be used, no matter what the Tg level is or the outcome of the ultrasound. In patients with negative Tg levels, no findings in the ultrasound of the neck as well as low Tg antibodies levels are classified as having an excellent response to the initial therapy. Their risk of recurrence is very low. For patients with an excellent response to the initial therapy, the ATA recommends ongoing monitoring with testing of non-stimulated Tg levels every 1–2 years and a neck ultrasound at 3–5-year intervals. Further imaging studies, such as a diagnostic WBS, an MRI, or an FDG-PET, are not deemed necessary (12).

There are some limitations resulting from the retrospective character of this study, particularly concerning the care of patients in the high-risk category. The need for a WBS in high-risk patients could further be studied in a prospective manner.

## Conclusion

In summary, this comprehensive two-center study, encompassing data from hospitals in two distinct countries, has uncovered a highly significant correlation between stimulated Tg levels and the outcomes of radioiodine WBS at the 4–12 months follow-up following ablation therapy. Our data indicate that routinely performing a radioiodine WBS on patients with low stimulated Tg levels (<0.5 ng/mL) may not be justified, given the rarity of positive results within this subgroup. Nevertheless, it is crucial to recognize that despite the low stimulated Tg levels, there have been instances of WBS-detected recurrence. Therefore, while the routine application of WBS may not be necessary for all patients with low stimulated Tg levels, more studies are needed to examine the necessity of the WBS as a component in follow-up protocols for high-risk patients.

## Supplementary Materials

Supplementary Material

## Declaration of interest

The authors declare that there is no conflict of interest that could be perceived as prejudicing the impartiality of the study reported.

## Funding

TS, NSL, HE, MH, AH, EKC, and GK received no funding.

## Author contribution statement

Conception and design: GK. Collection and assembly of data: TK, NSL. Data analysis: TK. Data interpretation: TK, NSL, HE, MH, AH, EK-C, GK. Article writing: TK. Final approval of article: TK, NSL, HE, MH, AH, EKC, GK. Responsible for integrity of the data and accuracy of the analysis: TK, NSL, HE, MH, AH, EKC, GK. TK and NSL are equal first coauthors of this article.
