# Supplementary material for: Value of follow-up diagnostic radioiodine scans in differentiated thyroid cancer
Source: Endocr Connect. 2024 Apr 12;13(5):e240007. doi: 10.1530/EC-24-0007 (PMC11046343; doi:10.1530/EC-24-0007)
Supplement: Supplementary Material [file supplementary_material.pdf]

Appendix

Supplementary information:

Break down of the patients TNM Staging

pT	Amount of pT	In %
1	26	7,03%
2	41	11,08%
3	89	24,05%
4	2	0,54%
1a	43	11,62%
1a2	1	0,27%
1am	24	6,49%
1b	68	18,38%
1b1	1	0,27%
1bm	14	3,78%
1m	15	4,05%
2a	5	1,35%
2b	4	1,08%
2m	6	1,62%
3b	7	1,89%
3bm	2	0,54%
3m	17	4,59%
4a	2	0,54%
4am	2	0,54%
c	1	0,27%
**Total**	**370**	**100,00%**

N	Amount of N	In %
0	157	42,43%
1	29	7,84%
1a	75	20,27%
1a1	1	0,27%
1b	57	15,41%
x	51	13,78%
**Total**	**370**	**100,00%**

M	Amount of M	In %
0	231	62,43%
x	135	36,49%
1	4	1,08%
**Total**	**370**	**100,00%**
